# Supplementary material for: Triaging HPV-Positive Cervical Samples with p16 and Ki-67 Dual Stained Cytology within an Organized Screening Program—A Prospective Observational Study from Western Norway
Source: Int J Mol Sci. 2023 Apr 12;24(8):7158. doi: 10.3390/ijms24087158 (PMC10138375; doi:10.3390/ijms24087158)
Supplement: Supplementary file 1 [file ijms-24-07158-s001.zip › ijms-2244007-supplementary.pdf]

**Supplemental Table S1**. Performance of CINtec PLUS as compared to cytology for triage of CIN2+ and CIN3+, alone or in combination with partial HPV genotyping in a cohort of primary hrHPV positive women with final diagnosis obtained within two years (n=1111).

| Follow-up endpoint | Triage strategy[1] | SE (95% CI) | SP (95% CI) | PPV (95% CI) | NPV (95% CI) | *p* values[2] |
|---|---|---|---|---|---|---|
| CIN2+ | CINtec PLUS | 84.2 (79.9, 87.8) n=330 | 63.8 (60.3, 67.1) n=781 | 49.6 (45.4, 53.7) n=561 | 90.5 (87.8, 92.7) n=550 | <0.001 (SE) 0.014 (SP) 0.92 (PPV) <0.001 (NPV) |
| | Cytology | 72.1 (67.0, 76.7) n=330 | 68.8 (65.4, 71.9) n=781 | 49.4 (44.9, 53.8) n=482 | 85.4 (82.4, 87.9) n=629 | |
| | HPV 16/18 | 50.6 (45.2, 56.0) n=330 | 78.6 (75.6, 81.3) n=781 | 50.0 (44.7, 55.3) n=334 | 79.0 (76.0, 81.7) n=777 | |
| | CINtec PLUS and HPV 16/18 | 46.4 (41.1, 51.8) n=330 | 91.7 (89.5, 93.4) n=781 | 70.2 (63.8, 75.9) n=218 | 80.2 (77.4, 82.7) n=893 | <0.001 (SE) 0.003 (SP) 0.22 (PPV) <0.001 (NPV) |
| | Cytology and HPV 16/18 | 36.7 (31.6, 42.0) n=330 | 94.5 (92.7, 95.9) n=781 | 73.8 (66.6, 79.9) n=164 | 77.9 (75.2, 80.5) n=947 | |
| | CINtec PLUS and/or HPV 16/18 | 88.5 (84.6, 91.5) n=330 | 50.7 (47.2, 54.2) n=781 | 43.1 (39.4, 46.9) n=677 | 91.2 (88.2, 93.6) n=434 | 0.19 (SE) 0.22 (SP) 0.69 (PPV) 0.32 (NPV) |
| | Cytology and/or HPV 16/18 | 86.1 (81.9, 89.4) n=330 | 52.9 (49.4, 56.4) n=781 | 43.6 (39.8, 47.4) n=652 | 90.0 (86.9, 92.4) n=459 | |
| CIN3 + | CINtec PLUS | 85.8 (81.3, 89.4) n=282 | 61.5 (58.2, 64.8) n=829 | 43.1 (39.1, 47.3) n=561 | 92.7 (90.2, 94.6) n=550 | <0.001 (SE) 0.004 (SP) 0.79 (PPV) 0.001 (NPV) |
| | Cytology | 74.5 (69.1, 79.2) n=282 | 67.2 (63.9, 70.3) n=829 | 43.6 (39.2, 48.0) n=482 | 88.4 (85.8, 90.8) n=629 | |
| | HPV 16/18 | 51.4 (45.6, 57.2) n=282 | 77.2 (74.2, 79.9) n=829 | 43.4 (38.2, 48.8) n=334 | 82.4 (79.5, 84.9) n=777 | |
| | CINtec PLUS and HPV 16/18 | 47.2 (41.4, 53.0) n=282 | 89.7 (87.5, 91.6) n=829 | 43.1 (39.1, 47.3) n=561 | 92.7 (90.2, 94.6) n=550 | <0.001 (SE) <0.001 (SP) 0.087 (PPV) 0.003 (NPV) |
| | Cytology and HPV 16/18 | 38.3 (32.8, 44.1) n=282 | 93.2 (91.3, 94.8) n=829 | 43.6 (39.2, 48.0) n=482 | 88.6 (85.8, 90.8) n=629 | |
| | CINtec PLUS and/or HPV 16/18 | 90.1 (86.0, 93.0) n=282 | 49.0 (45.6, 52.4) n=829 | 43.4 (38.2, 48.8) n=334 | 82.4 (79.5, 84.9) n=777 | 0.21 (SE) 0.21 (SP) 0.70 (PPV) 0.32 (NPV) |
| | Cytology and/or HPV 16/18 | 87.6 (83.2, 90.9) n=282 | 51.1 (47.7, 54.5) n=829 | 61.0 (54.4, 67.2) n=218 | 83.3 (80.7, 85.6) n=893 | |

[1] Triage strategies defined in Box1, [2] *p* values for comparison of triage involving cytology vs. triage involving CINtec PLUS.
Abbreviations: SE (Sensitivity), SP (Specificity), PPV (Positive predictive value), NPV (Negative predictive value), hrHPV (high risk HPV).
